# Detection of Circulating Tumor Cell Molecular Subtype in Pulmonary Vein Predicting Prognosis of Stage I–III Non-small Cell Lung Cancer Patients

**DOI:** 10.3389/fonc.2019.01139

**Published:** 2019-10-29

**Authors:** Jingsi Dong, Daxing Zhu, Xiaojun Tang, Xiaoming Qiu, Dan Lu, Bingjie Li, Dan Lin, Qinghua Zhou

**Affiliations:** Department of Lung Cancer Center, West China Hospital, Sichuan University, Chengdu, China

**Keywords:** pulmonary vein, CTC, non-small cell lung cancer, PD-L1, EMT

## Abstract

**Background:** There was rare studies on prognosis of pulmonary venous CTC and early or advanced NSCLC patients. We want to investigate whether CTCs and the subtype of it can predict the prognosis of NSCLC patients.

**Patients and Methods:** One hundred and fourteen patients with stage I-III NSCLC were included CanPatrol™ CTC analysis. PD-L1 expression level were detected in CTC of pulmonary vein. PD-L1, number of CTC in pulmonary, CTC's subtype, clinical characteristics, prognosis of patients were analyzed.

**Results:** 110/114 (96.5%) patients could be found CTCs in pulmonary vein, 58/114 (50.9%) patients had CTC≥15/ml in pulmonary vein, 53/110 patients (48.2%) were defined as having MCTC subtype and 56/110 patient were found have PD-L1 (+) CTC in pulmonary vein. Multivariate analyses showed that PVCTC, MCTC, and stage were independent factors of DFS (*P* < 0.05). No OS difference was found between number of CTC (*P* = 0.33) and other CTC factors (*P* > 0.05), only stage was independent factor of OS (*P* = 0.019). There were decreases of CTC number and MCTC number in EGFR mutant subgroup (*P* = 0.0009 and *P* = 0.007). There were increases of CTC (*P* = 0.0217), MCTC (*P* = 0.0041), and PD-L1 (+) CTC (*P* = 0.0002) number in KRAS mutant subgroup. There was increase of MCTC (*P* =0.0323) number in BRAF mutant. There were fewer CTCs in pulmonary vein for patients with EGFR mutant than in patients with full wild-type gene (*P* = 0.0346). There were more PD-L1 positive CTCs in pulmonary vein for patients with ALK rearrangement, KRAS mutant, BRAF mutant, or ROS1 mutant than in patients with full wild-type gene (*P* = 0.0610, *P* = 0.0003, *P* = 0.032, and *P* = 0.0237). There were more mesenchymal CTCs in pulmonary vein for patients with KRAS mutant and BRAF mutant than in patients with full wild-type gene (*P* = 0.073 and *P* = 0.0381). There were fewer mesenchymal CTCs in pulmonary vein for patients with EGFR mutant than in patients with full wild-type gene (*P* = 0.0898).

**Conclusions:** The patients with high number of CTCs, MCTCs, or PD-L1 (+) CTCs in pulmonary vein experienced poor prognosis of DFS. There are obvious correlations between the CTC subtype of NSCLC and the gene subgroups of tumor tissue.

## Introduction

Lung cancer, known as a public health problem in the world, is the leading cause of death caused by malignant tumors worldwide. According to Cancer Statistics published in CA, the estimated deaths caused by lung cancer in 2018 number 83,550 for males and 70,500 for females (1). In China, Lung cancer is the leading cause of death of male and female malignancies (2). Non-small cell lung cancer (NSCLC) accounts for 85% of all lung cancers, and post-treatment recurrence and metastasis are the leading cause of death. Despite many advances in treatment, the overall 5-year survival rate for lung cancer is <20% (3).

In 2002, Dunn et al. proposed the theory of immune editing (4). In recent years, pd-l1 inhibitors have been approved for the treatment of advanced non-small cell lung cancer and achieved remarkable results (5, 6). At present, the recognized detection of programmed cell death ligand 1 (PD-L1) was still at the level of tissue samples, and there was rear research attention to PD-L1 detection of circulating tumor cell (CTC), which was the root of tumor metastasis. Many clinical studies on the CTCs of NSCLC have shown the reliability of CTCs as a prognostic indicator (7–9). A study of Europe confirm CTCs as an independent prognostic indicator of progression-free survival and overall survival in advanced NSCLC (10). Wang's study indicates that CTC detection is mainly related to tumor stage, lymph node metastasis and prognosis, and CTC detection is significantly associated with the shortening of progression-free survival (PFS) and overall survival (OS) in NSCLC (11). CTCs had already been considered the leading causes and markers for tumor recurrence and metastasis (7). Study on the relationship between postoperative disease-free survival (DFS) and CTC in pulmonary vein of NSCLC patient is rear. A study showed that CTCs isolated from early stages of lung cancer are predictive of poor prognosis and can be interrogated to determine biomarkers predictive of recurrence (12), but this study included only 36 patient of lung cancer (NSCLC = 35, SCLC = 1) and the stages of patients was not all early (19 patients were stage I, seven were stage II, eight were stage III, and one patient was stage IV disease).

During the dissemination of cancer cells, epithelial cells frequently exhibit a downregulation of epithelial markers and a loss of intercellular junctions (13). The loss of epithelial features is often accompanied by increased expression of mesenchymal genes. This process, described as epithelial-mesenchymal transition (EMT), endows cancer cells with migratory and invasive properties and promotes cancer recurrence (14–16). Although the number of CTC in pulmonary veins is the largest, current studies have not focused on the relationship between molecular subtypes of CTC in pulmonary and prognosis of cancer therapy.

Here in this study, we focused on the relationship between pulmonary venous CTCs (including different CTC molecular subtypes) and postoperative prognosis of patients with stage I-III NSCLC. The relationship between different CTC molecular subtypes in pulmonary veins and tumor molecular subgroups (EGFR, KRAS, ALK, and BRAF) was studied by translational medicine methods.

## Methods

### Study Design

**Figure 1:**
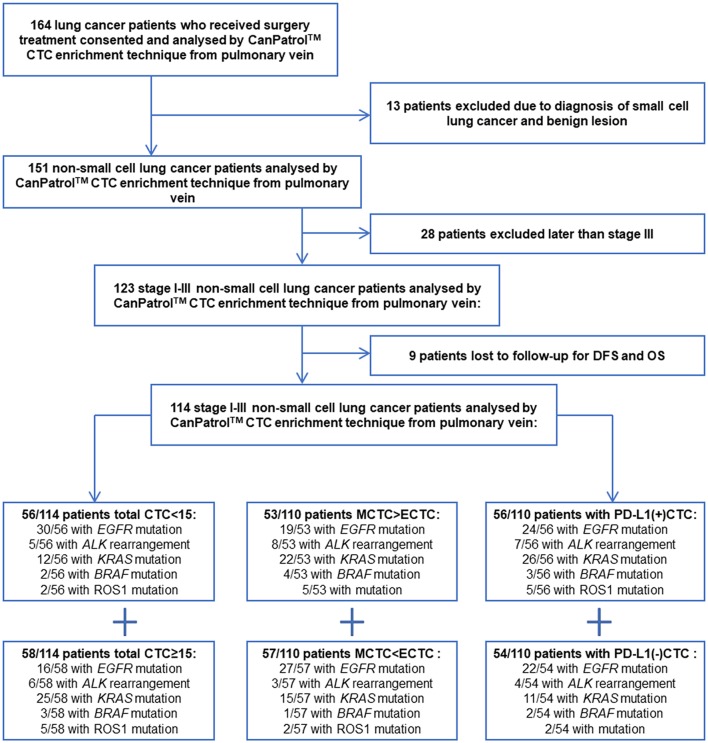
Patient flow.

One hundred and sixty-four non-small cell lung cancer (NSCLC) patients with stage I-III who could receive surgical resection at the West China Hospital of Sichuan University were included in this study from February 2017 to January 2019. One hundred and fourteen patients eventually met the inclusion criteria. The study was approved by the medical ethics committee of Sichuan University. The patient flow is show in Figure 1. All the patients were informed of the procedure and signed informed consent. Our report adheres to the REMARK criteria (17). Inclusion criteria: (a) NSCLC patients who received surgery in Lung Cancer Center of West China Hospital; (b) age of more than 18 years old; (c) postoperative pathological stages were stage I to III; (d) patients have complete clinical data and follow-up data.

In this study, all of the patients with lung cancer received conventional thoracotomy. During surgery, the roots of the pulmonary vein were ligated at the proximal end of the heart, and then 5 ml of blood was extracted at the distal end of the pulmonary vein which was shown in Figure S1. After the blood was extracted from the pulmonary vein, it was immediately injected into the blood vessel containing EDTA. Next, the proximal end of the pulmonary vein was ligated a second time, and the distal end of the pulmonary vein was ligated finally (Figure S1). Routine laboratory analyses were also performed on all patients, with data prospectively collected for age, sex, histological subtype, genotype, ECOG performance status, smoking status, sites of metastasis, treatment received, stage, date of progression, date of death as the previous studies have been published (18).

Mutations in EGFR exons 18 through 21 were examined using a DxS EGFR mutation test kit (Amoy Diagnostics, China). KRAS mutation was analyzed by Sanger sequencing as described. ALK rearrangement was detected by FISH using the Vysis LSI ALK Break Apart FISH Probe (Abbott Molecular, USA) according to the manufacturer's instructions as described (19). Somatic mutation analysis of BRAF and ROS1 was analyzed by SurPlex-xTAG70plex (Surexam, China).

### CTC Analysis

**Figure 2:**
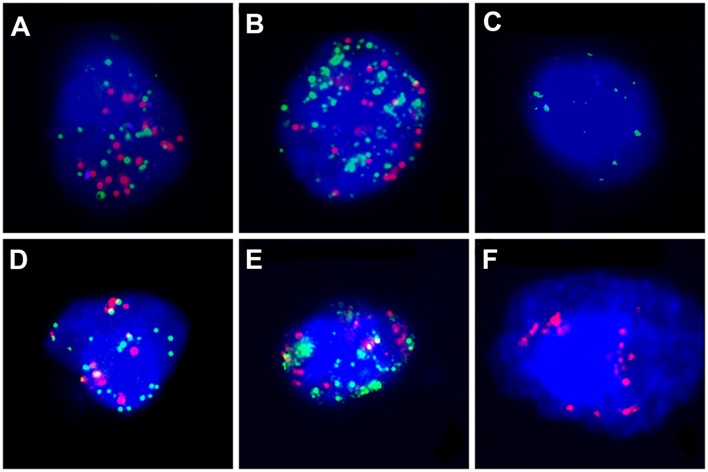
The PD-L1 mRNA expression level and CTC subtypes in CTCs was detected by RNA-ISH. PD-L1: purple fluorescence, epithelial markers: red fluorescence, mesenchymal markers: green fluorescence. **(A)** Hybrid type CTC with PD-L1(++); **(B)** Hybrid type CTC with PD-L1(+); **(C)** Mesenchymal Type CTC with PD-L1(–); **(D)** Mixed type CTC with PD-L1(+); **(E)** Hybrid type CTC with PD-L1(–); **(F)** Epithelial type CTC with PD-L1(–).

CanPatrol™ (Surexam Biotech, Guangzhou, China) was used to identify CTCs in lung adenocarcinoma patients, as previously described (Figure S2). PD-L1, EpCAM, CK8, CK18, and CK19, vimentin, and twist gene expression levels from these different cell types were also detected by RNA *in situ* hybridization. The detection method of CTC has been described in detail in our published articles (20). The above markers were used to help distinguish among epithelial, mesenchymal, and hybrid phenotype CTCs. Detection and classification of CTCs using multiple epithelial markers, including EpCAM, CK8/18/19 (red fluorescence) and mesenchymal markers such as Vimentin and Twist (green fluorescence) which were shown in Figure 2. The PD-L1 mRNA expression level in CTCs was detected by RNA-ISH (purple fluorescence) which were shown in Figure 2.

### Statistical Analysis

REMARK guidelines were followed in planning, analysis and reporting of this study. SPSS Statistics 19 software (IBM Deutschland GmbH, Germany) was used for statistical analysis. A *P* < 0.05 was considered a statistically significant difference. GraphPad Prism 6.02 was used for image processing. The survival curve of the OS and DFS of NSCLC patients were plotted by the Kaplan–Meier method after the log-rank test. OS was the time from surgery to death. DFS was the time from surgery to the time of diagnosis of local recurrence, distant metastasis or death, whichever occurred first. The Cox regression model was used for multivariate analysis of all independent influence factors, including the CTC results and other factors, on OS and DFS. Kaplan–Meier curves were computed using GraphPad Prism 6.02. *T*-test was used to compare and analyze continuous variable factors in this study. Chi-squared test was used to analyze the factors of categorical variables. Significance was indicated by the *P*-values of two-tailed tests <0.05.

## Results

### Patient Characteristics

One hundred and fourteen patients were included in this study finally as shown in Figure 1. Forty-nine patients were female (42.98%), and 65 were male (57.02%). Forty squamous lung cancer patients (35.08%) and 68 patients with adenocarcinoma lung cancer (59.65%) were included in this study, only six patients with other histological types of NSCLC. Fifty-one stage I patients, 21 stage II patients, and 42 stage III patients were included in this study.

CTCs were found in 110 patients' pulmonary veins, only four patients with no CTC can be found. According to the median number of CTC in patients' pulmonary veins, patients were divided into group with CTC≥15 (58/114, 50.9%) and group with CTC<15 (56/114, 49.1%). The Baseline Clinical characteristics was shown in Table 1. According to the CTC subtype in patients' pulmonary veins, patients were divided into group with mesenchymal CTC (53/110, 48.2%) and group with non- mesenchymal CTC (epithelial and hybrid subtypes, 57/110, 51.8%). The Baseline Clinical characteristics was shown in Table S1. According to whether express PD-L1 in CTC of patients' pulmonary veins or not, patients were divided into group with PD-L1 positive CTC (56/110, 50.1%) and group with PD-L1 negative CTC (54/110, 49.9%). The Baseline Clinical characteristics was shown in Table S2.

**Table 1:** Baseline clinical characteristics and CTC status of enrolled non-small cell patients.

**Characteristic**	**Total CTC <15 (%)**	**Total CTC≥15 (%)**	***P*-value**
Total patient numbers	56/114 (49.1)	58/114 (50.9)	0.791
Age (mean)	59.7	59.2	0.931
Gender			0.685
Female	23/56 (41.1)	26/58 (44.8)	
Male	33/56 (58.9)	32/58 (55.2)	
Smoking status (piece^*^year)	317.9	432.7	
Histology			
Squamous	13/56 (23.2)	15/58 (25.9)	0.743
Adenocarcinoma	42/56 (75.0)	41/58 (70.7)	0.605
Others^*^	1/56 (1.8)	2/58 (3.4)	0.579
Surgical method			
Lobectomy	39/56 (69.6)	41/58 (70.7)	0.903
Segmentectomy	12/56 (21.4)	7/58 (12.1)	0.180
Sleeve lobectomy	4/56 (7.1)	8/58 (13.8)	0.247
Pneumonectomy	1/56 (1.8)	2/58 (3.4)	0.579
Stage (AJCC 8)			0.361
Stage I-II	42/56 (75.0)	39/58 (67.2)	
Stage III	14/56 (25.0)	19/58 (32.8)	
Performance status (EGOG)			0.262
0–1	54/56 (96.4)	53/58 (91.4)	
2	2/56 (3.6)	5/58 (8.6)	
Adjuvant chemotherapy			0.420
Yes	20/56 (35.7)	25/58 (43.1)	
No	36/56 (64.3)	33/58 (56.9)	
Adjuvant radiotherapy			0.216
Yes	10/56 (17.9)	16/58 (27.6)	
No	46/56 (82.1)	42/58 (72.4)	

* *Large cell carcinoma*.

### Clinical Relevance of Total CTCs and CTC Subtype

**Figure 3:**
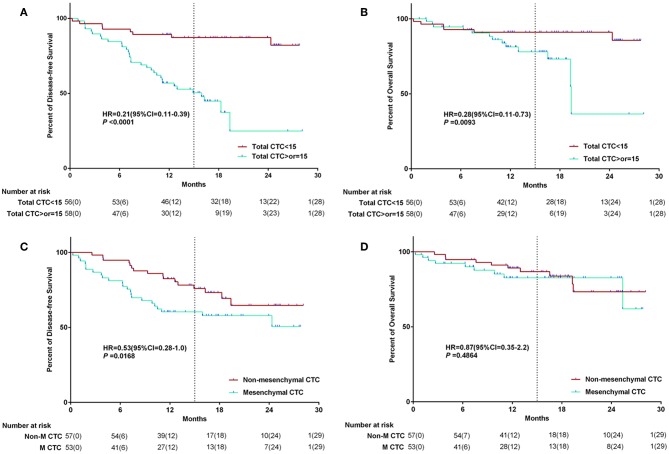
The Kaplan–Meier survival curve of CTC number and MCTC number association with survival. The relationship between CTC quantity and survival **(A,B)**; The relationship between CTC subtype and survival **(C,D)**.

The median DFS time of the whole group was 20.6 (CI: 18.7–22.5) months (follow-up range: 0–30 months). The median overall survival time of the whole group was 24.3 (CI: 22.5–25.8) months (follow up range: 0–30 months). After follow-up, univariate analyses showed significant reductions in median DFS in CTCs≥15 patients group. The median postoperative DFS was 15.3 (CI: 12.3–18.4) months (range: 0–30 months) in the group with pulmonary vein CTCs≥15 patients, and 24.7 (CI: 22.7–26.7) months (range: 0–30 months) in the group with pulmonary vein CTCs<15 patients (*P* < 0.001). The median postoperative overall survival (OS) was 20.2 (CI: 16.3–24.1) months (range: 0–30 months) in the group with pulmonary vein CTCs≥15 patients, and 25.4 (CI: 23.6–27.3) months (range: 0–24 months) in the group with pulmonary vein CTCs<15 patients (*P* = 0.0093). And there was significant reductions in median DFS in MCTC patients group. The median postoperative DFS was 18.4 (CI: 15.4–21.4) months in the MCTC group, and 22.5 (CI: 20.2–24.8) months in the Non-MCTC group (*P* = 0.0168). And there was no significant difference in OS between the two groups of CTC subtype (*P* = 0.4864). The Kaplan–Meier survival curve of above data was shown in Figure 3.

### Clinical Relevance of PD-L1 Expression in CTCs and Stage of Patients

After follow-up, univariate analyses showed significant reductions in median DFS in PD-L1 positive CTC patients group. The median postoperative DFS was 16.7 (CI: 13.9–19.3) months (range: 0–30 months) in the group with PD-L1 positive CTC, while 24.5 (CI: 22.4–26.5) months (range: 0–30 months) in the group with PD-L1 negative CTC (*P* = 0.0003). There was no significant difference in OS between the two groups (*P* = 0.09).

**Figure 4:**
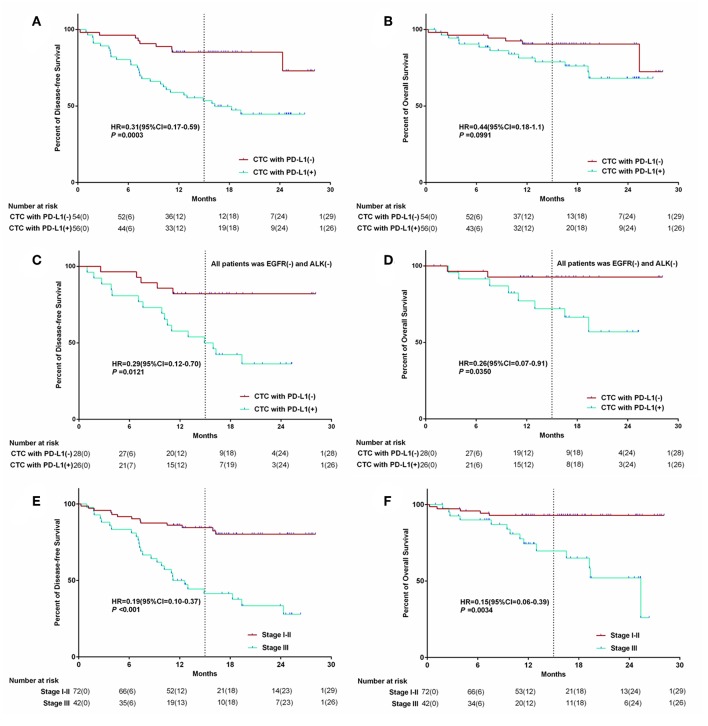
The Kaplan–Meier survival curve of PD-L1 (+) CTC number and stage of patient association with survival. The relationship between PD-L1 expreesion in CTC and survival **(A,B)**; The relationship between PD-L1 expreesion in CTC for the wild type EGFR&ALK patients and survival **(C,D)**; The relationship between Stage and survival **(E,F)**.

For the group of EGFR negative and ALK negative, there were also significant reductions in median postoperative DFS and OS in PD-L1 positive CTC patients group (showed in Figures 4C,D, *P* < 0.05). And there was significant reductions in median DFS and OS in stage I-II patients group. The median postoperative DFS was 24.1 (CI: 22.1–26.1) months in the stage I-II patients group, and 14.7 (CI: 11.8–17.5) months in the stage III group (*P* < 0.001). The median postoperative overall survival (OS) was 26.4 (CI: 24.9–27.9) months in the stage I-II patients group, and 19.1 (CI: 16.0–22.0) months in the stage III group (*P* = 0.034). The Kaplan–Meier survival curve of above data was shown in Figure 4.

In multivariate survival analysis, patients' clinical data, tumor's gene type, number of pulmonary venous CTC, and CTC subtype were included stage, mesenchymal CTCs, and the number of pulmonary vein CTCs were the independent factors of DFS, and only stage was independent factors of OS (showed in Table S3).

### Relationship Between CTC Subtype and NSCLC Gene Subgroup

All of the 114 patients received gene test after surgery using the tissue samples. 46/114 (40.4%) patients have found EGFR mutations, 11/114 (9.6%) patients have found ALK rearrangements, 37/114 (32.5%) patients have found KRAS mutations and 5/114 (4.4%) patients have found BRAF mutations. As shown in Table 2, for the patient with PVCTC≥1, there was 53 of 114 patients with MCTC>ECTC, there was 56/114 patients with PD-L1 (+) CTC. For the patient with PVCTC≥15, there was 26 of 114 patients with MCTC>ECTC, there was 24/114 patients with PD-L1 (+) CTC.

**Table 2:** Baseline CTC characteristics of patients with advanced NSCLC according to total, EMT and PD-L1+ CTC status.

**Group**	***N* (%) total = 114**
No. of patient with no CTC in PV	4/114 (0.035)
No. of patient with CTC≥1 in PV	
Total	110/114 (0.965)
MCTC>ECTC	53/114 (46.5)
PD-L1+CTC	56/114 (49.1)
MCTC>ECTC with PD-L1+CTC	27/114 (23.7)
No. of patient with CTC≥15 in PV	58/114 (50.9)
MCTC>ECTC	26/114 (22.8)
PD-L1+CTC	24/114 (21.1)
MCTC>ECTC with PD-L1+CTC	9/114 (7.9)

**Figure 5:**
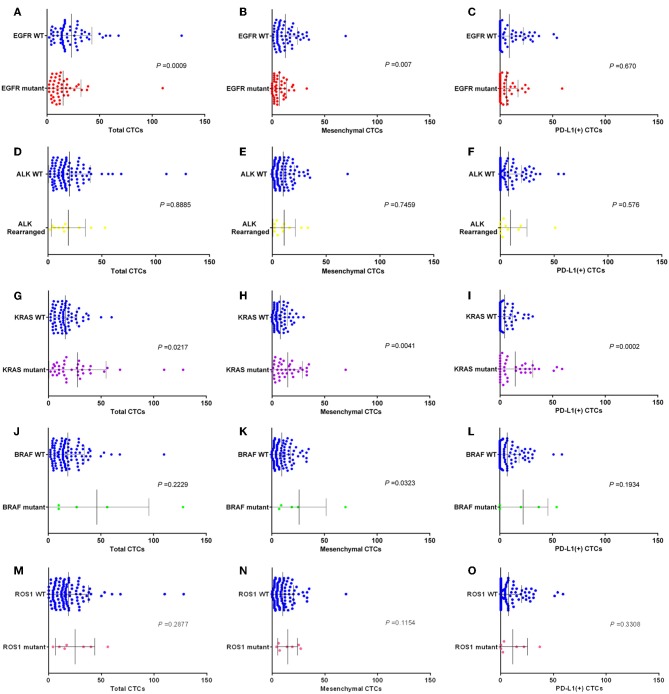
Box plots assessing differences in relative numbers of total CTCs, MCTCs, and PD-L1 (+) CTCs in EGFR. **(A–C)**, ALK **(D–F)**, KRAS **(G–I)**, BRAF **(J–L)**, and ROS1 **(M–O)** subgroups. *P-*values obtained by Mann–Whitney tests.

For the detection of the EGFR mutant subgroup, there were fewer CTCs in pulmonary vein for patients with EGFR mutant than in patients with wild-type EGFR (EGFR mutant vs. WT: mean 15.3 vs. 23.2, *P* = 0.0009). There were fewer mesenchymal CTCs in pulmonary vein for patients with EGFR mutant than in patients with wild-type EGFR (EGFR mutant vs. WT: mean 6.9 vs. 12.6, *P* = 0.0007). And there were little fewer PD-L1 positive CTCs in pulmonary vein for patients with EGFR mutant than in patients with wild-type EGFR, but the difference was not statistically significant (EGFR mutant vs. WT: median 6.4 vs. 8.8, *P* = 0.67). The data above was shown in Figures 5A–C.

For the detection of the ALK rearrangement subgroup, there was no difference of CTCs number in pulmonary vein between patients with ALK rearrangement and patients with wild-type ALK (ALK rearrangement vs. WT: mean 16.8 vs. 19.9, *P* = 0.8885). There was no difference of mesenchymal CTCs number in pulmonary vein between patients with ALK rearrangement and patients with wild-type ALK (ALK rearrangement vs. WT: mean 9.4 vs. 10.1, *P* = 0.7459). And there was also no difference of PD-L1 positive CTCs number in pulmonary vein between patients with ALK rearrangement and patients with wild-type ALK (ALK rearrangement vs. WT: mean 10.1 vs. 7.6, *P* = 0.576). The data above was shown in Figures 5D–F.

For the detection of the KRAS mutant subgroup, there were more CTCs in pulmonary vein for patients with KRAS mutant than in patients with wild-type KRAS (KRAS mutant vs. WT: mean 28.1 vs. 16.0, *P* = 0.0217). There were more mesenchymal CTCs in pulmonary vein for patients with KRAS mutant than in patients with wild-type KRAS (KRAS mutant vs. WT: mean 15.2 vs. 7.8, *P* = 0.0041). And there were more PD-L1 positive CTCs in pulmonary vein for patients with KRAS mutant than in patients with wild-type KRAS (KRAS mutant vs. WT: mean 14.8 vs. 4.4, *P* = 0.0002). The data above was shown in Figures 5G–I.

For the detection of the BRAF mutant subgroup, there were more CTCs in pulmonary vein for patients with BRAF mutant than in patients with wild-type BRAF, but the difference was not statistically significant (BRAF mutant vs. WT: mean 55.2 vs. 18.6, *P* = 0.2229). There were more mesenchymal CTCs in pulmonary vein for patients with BRAF mutant than in patients with wild-type BRAF, but the difference was not statistically significant (BRAF mutant vs. WT: mean 30.8 vs. 9.5 *P* = 0.0323). And there were more PD-L1 positive CTCs in pulmonary vein for patients with BRAF mutant than in patients with wild-type BRAF, but the difference was not statistically significant (BRAF mutant vs. WT: mean 27.7 vs. 7.5, *P* = 0.1934). The data above was shown in Figures 5J–L.

For the detection of the ROS1 mutant subgroup, there were more CTCs in pulmonary vein for patients with ROS1 mutant than in patients with wild-type ROS1, but the difference was not statistically significant (ROS1 mutant vs. WT: mean 25.0 vs. 18.7, *P* = 0.2877). There were more mesenchymal CTCs in pulmonary vein for patients with ROS1 mutant than in patients with wild-type ROS1, but the difference was not statistically significant (ROS1 mutant vs. WT: mean 14.5 vs. 9.9, *P* = 0.1154). And there were more PD-L1 positive CTCs in pulmonary vein for patients with ROS1 mutant than in patients with wild-type ROS1, but the difference was not statistically significant (ROS1 mutant vs. WT: mean 11.3 vs. 7.4, *P* = 0.1934). The data above was shown in Figures 5M–O.

**Figure 6:**
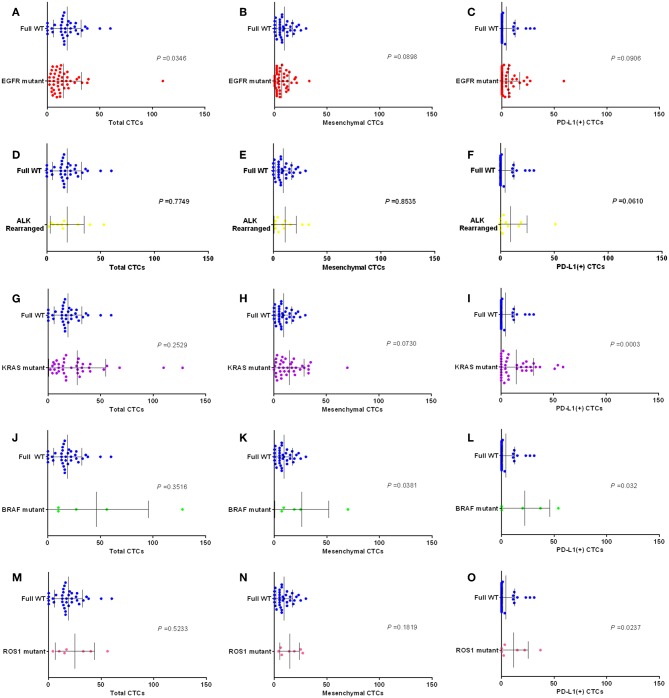
Box plots assessing differences in relative numbers of total CTCs, MCTCs and PD-L1 (+) CTCs in EGFR **(A–C)**, ALK **(D–F)**, KRAS **(G–I)**, BRAF **(J–L)**, and ROS1 **(M–O)** subgroups (mutant vs. full WT). *P-*values obtained by Mann–Whitney tests.

Furthermore, we compared the each molecular subgroup with full WT group. As shown in Figure 6, for the detection of the EGFR mutant subgroup, there were fewer CTCs in pulmonary vein for patients with EGFR mutant than in patients with full wild-type gene (*P* = 0.0346); There were more PD-L1 positive CTCs in pulmonary vein for patients with ALK rearrangement, KRAS mutant, BRAF mutant, or ROS1 mutant than in patients with full wild-type gene (*P* = 0.0610, *P* = 0.0003, *P* = 0.032, and *P* = 0.0237); There were more mesenchymal CTCs in pulmonary vein for patients with KRAS mutant and BRAF mutant than in patients with full wild-type gene (*P* = 0.073 and *P* = 0.0381); There were fewer mesenchymal CTCs in pulmonary vein for patients with EGFR mutant than in patients with full wild-type gene (*P* = 0.0898).

## Discussion

Pulmonary veins are the closest reflux vessels to tumors. Pulmonary veins are the main route for tumor cells to enter the blood from tumor tissues of NSCLC. Okumura et al. found that CTCs in the pulmonary vein were significantly higher than those in peripheral blood (21). Lindsay et al. (18) had reported that PFS and OS are shorter in patients with advanced non-small cell lung cancer whose peripheral venous CTC is >5/ml. A study showed that CTC monitoring after SBRT for presumed early stage NSCLC may give lead-time notice of disease recurrence or progression (22). A study in 2012 demonstrated that CTC test has high sensitivity in early and advanced lung cancer (23). So far, studies that have focused entirely on the relationship between CTC and survival in patients with early non-small cell lung cancer have not been published. In our point of view, the number of tumor cells in peripheral venous blood is not accurate because peripheral blood first flows through tissue cells and then flows back into venous blood. In this study, we confirmed that the patients with the number of CTC≥15/5 ml in pulmonary veins had the significant shorter postoperative DFS (Figure 3A, *P* < 0.0001). Although patients had received multiple comprehensive treatments after postoperative relapse, the OS of patients included in this study was relatively determined by the amount of CTC in pulmonary veins (Figure 3B, *P* = 0.0093). All the patients enrolled in this study underwent open-chest surgery, while there was no patient received thoracoscopic surgery. The reason is that open-chest surgery is appropriate for NSCLC patients with stage I to III, while thoracoscopic surgery is commonly considered appropriate for NSCLC patients with stage I to II all over the world.

Although the Cellsearch System has been used in the majority of published studies, it depends on tumor epithelial cell expression of EpCAM, the presence of an intact nucleus, and the absence of CD45 (4, 7, 25–31) (16, 18). However, this method lacks the detection of mesenchymal CTC, and the EMT of CTC is easy to be ignored. Recently, Canpatrol™ CTC analysis system was developed to detect CTC and classify EMT phenotypes via multiple mRNA *in situ* hybridization assay, by which revealing that CTC count and EMT classification are correlated with clinical stage and prognosis in many kinds of cancers (24, 25). In this study, we found that postoperative DFS was shorter in patients with mesenchymal CTC predominance than in patients with epithelial and hybrid type (Figure 3C, *P* = 0.0168). Different from the effect of tumor cell count on OS, the OS of patients included in this study was not completely determined by subtype of CTC in pulmonary veins (Figure 3D, *P* = 0.48).

The PD-L1 will downregulate T-cell activation and promote immune escape when binding with programmed death 1 (PD-1) protein expressed on the T-cell surface (26). Many studies have found that the expression of PD-L1 in tumors is significantly correlated with the treatment response of nivolumab (27, 28). PD-L1 expression correlated with benefit to immune checkpoint inhibitors, not only nivolumab. Although there were some differences reported in the results of clinical trials in which different PD-1/PD-L1 inhibitors were involved, the benefit of immunotherapy was significantly higher in those with high expression of PD-L1 than in those with low expression (29, 30). Until now, there is no study on detection of PD-L1 in CTC has been performed expect us. Detection of expression of PD-L1 in CTC will be an important supplement to liquid biopsy and of great significance to guide the treatment of cancer. In this study, we not only successfully detected the expression of PD-L1 in CTC, but also found that whether the express of PD-L1 or not was correlated with postoperative DFS and OS in NSCLC patients (Figure 4A, *P* = 0.0003; Figure 4B, *P* = 0.0991). For the patients with EGFR(–) and ALK(–), the effect of PD-L1 positive in CTC to survival (DFS & OS) was more obvious, as shown in Figures 4C,D. This phenomenon may be related to PD-L1-mediated tumor immune avoidance in NSCLC. It is well-known that UICC stage is an important factor determining the prognosis of patients in NSCLC. In this study, we confirmed that postoperative pathological stage of NSCLC patients is obviously related to postoperative DFS and OS (Figures 4E,F, *P* < 0.05).

The growth of NSCLC is related to many driver genes, including EGFR, KRAS, BRAF, ALK, and so on. NSCLC tumors have different genetic subgroups, even with the same pathological type (29, 31). In this study, we found that there were differences in the number of CTC, the number of mesenchymal CTC, and the number of PD-L1 positive CTC among patients with different NSCLC genotypes (Figure 5). The relationship between information of PD-L1 in tissue and PD-L1 in CTC was published by us in previous study (20). And we found that positive PD-L1 in CTC was positively correlated with positive PD-L1 in tissues. So in this study, we included new group of patients to do many more survival analyses and focus on the relationship between information of PD-L1 in CTC and gene type of tissues. Within tumor cells, the network of multiple driver genes plays an important role in tumor growth and immune escape. This just shows that different genotypes of tumor surface lead to different types of CTC into the blood.

## Conclusions

In conclusion, we found that pulmonary venous examination is a more reliable method for analyzing CTC in NSCLC patients receiving surgical treatment. In addition, the detection of PD-L1 expression in CTC may provide an important decision for post-operative adjuvant immunotherapy. In subsequent studies, we will focus on the impact of different conditions of CTC on postoperative adjuvant therapy effect, so as to provide more information for individualized treatment.

## Data Availability Statement

All datasets for this study are included in the manuscript/Supplementary Material.

## Ethics Statement

The studies involving human participants were reviewed and approved by the medical ethics committee of Sichuan University. The patients/participants provided their written informed consent to participate in this study.

## Author Contributions

JD and QZ designed the study. JD, BL, and DLi wrote the manuscript. DZ and XT supported the methods of the study. XQ and DLu supported the literature and statistical methods.

### Conflict of Interest

The authors declare that the research was conducted in the absence of any commercial or financial relationships that could be construed as a potential conflict of interest.

## Abbreviations

CTC: circulating tumor cell
NSCLC: non-small cell lung cancer
EMT: epithelial-mesenchymal transition
WT: wild-type
MCTC: mesenchymal circulating tumor cell
ECTC: epithelial circulating tumor cell
PVCTC: pulmonary vein circulating tumor cell
PFS: progression-free survival
DFS: disease-free survival
OS: overall survival
PD-L1: programmed cell death ligand 1
EGFR: epidermal growth factor receptor
KRAS: kirsten rat sarcoma viral oncogene
BRAF: v-raf murine sarcoma viral oncogene homolog B
ALK: anaplastic lymphoma kinase.

## References

[1] Siegel RL Miller KD Jemal A Cancer statistics, 2018. CA Cancer J Clin. (2018) 68:7–30. 10.3322/caac.21442 29313949

[2] Chen W Zheng R Baade PD Zhang S Zeng H Bray F. Cancer statistics in China, 2015. CA Cancer J Clin. (2016) 66:115–32. 10.3322/caac.21338 26808342

[3] Torre LA Siegel RL Jemal A. Lung cancer statistics. Adv Exp Med Biol. (2016) 893:1–19. 10.1007/978-3-319-24223-1_1 26667336

[4] Dunn GP Bruce AT Ikeda H Old LJ Schreiber RD. Cancer immunoediting: from immunosurveillance to tumor escape. Nat Immunol. (2002) 3:991–8. 10.1038/ni1102-991 12407406

[5] Giroux Leprieur E Dumenil C Julie C Giraud V Dumoulin J Labrune S. Immunotherapy revolutionises non-small-cell lung cancer therapy: results, perspectives and new challenges. Eur J Cancer. (2017) 78:16–23. 10.1016/j.ejca.2016.12.041 28407528

[6] Gandhi L Rodriguez-Abreu D Gadgeel S Esteban E Felip E De Angelis F. Pembrolizumab plus chemotherapy in metastatic non-small-cell lung cancer. N Engl J Med. (2018) 378:2078–92. 10.1056/NEJMoa1801005 29658856

[7] Krebs MG Sloane R Priest L Lancashire L Hou JM Greystoke A. Evaluation and prognostic significance of circulating tumor cells in patients with non-small-cell lung cancer. J Clin Oncol. (2011) 29:1556–63. 10.1200/JCO.2010.28.7045 21422424

[8] Aggarwal C Wang X Ranganathan A Torigian D Troxel A Evans T. Circulating tumor cells as a predictive biomarker in patients with small cell lung cancer undergoing chemotherapy. Lung Cancer. (2017) 112:118–25. 10.1016/j.lungcan.2017.08.008 29191584

[9] Syrigos K Fiste O Charpidou A Grapsa D. Circulating tumor cells count as a predictor of survival in lung cancer. Crit Rev Oncol Hematol. (2018) 125:60–8. 10.1016/j.critrevonc.2018.03.004 29650278

[10] Lindsay CR Blackhall FH Carmel A Fernandez-Gutierrez F Gazzaniga P Groen HJM. EPAC-lung: pooled analysis of circulating tumour cells in advanced non-small cell lung cancer. Eur J Cancer. (2019) 117:60–8. 10.1016/j.ejca.2019.04.019 31254940

[11] Wang X Ma K Yang Z Cui J He H Hoffman AR. Systematic correlation analyses of circulating tumor cells with clinical variables and tumor markers in lung cancer patients. J Cancer. (2017) 8:3099–104. 10.7150/jca.18032 28928901 PMC5604461

[12] Murlidhar V Reddy RM Fouladdel S Zhao L Ishikawa MK Grabauskiene S. Poor prognosis indicated by venous circulating tumor cell clusters in early-stage lung cancers. Cancer Res. (2017) 77:5194–206. 10.1158/0008-5472.CAN-16-2072 28716896 PMC5600850

[13] Chen Y Li S Li W Yang R Zhang X Ye Y Circulating tumor cells undergoing EMT are poorly correlated with clinical stages or predictive of recurrence in hepatocellular carcinoma. Sci Rep. (2019) 9:7084 10.1038/s41598-019-43572-1 31068623 PMC6506548

[14] Kalluri R Weinberg RA. The basics of epithelial-mesenchymal transition. J Clin Investig. (2009) 119:1420–8. 10.1172/JCI39104 19487818 PMC2689101

[15] Thiery JP Acloque H Huang RY Nieto MA. Epithelial-mesenchymal transitions in development and disease. Cell. (2009) 139:871–90. 10.1016/j.cell.2009.11.007 19945376

[16] Qi LN Xiang BD Wu FX Ye JZ Zhong JH Wang YY. Circulating tumor cells undergoing EMT provide a metric for diagnosis and prognosis of patients with hepatocellular carcinoma. Cancer Res. (2018) 78:4731–44. 10.1158/0008-5472.CAN-17-2459 29915159

[17] Sauerbrei W Taube SE McShane LM Cavenagh MM Altman DG. Reporting recommendations for tumor marker prognostic studies (REMARK): an abridged explanation and elaboration. J Natl Cancer Inst. (2018) 110:803–11. 10.1093/jnci/djy088 29873743 PMC6093349

[18] Lindsay CR Faugeroux V Michiels S Pailler E Facchinetti F Ou D. A prospective examination of circulating tumor cell profiles in non-small-cell lung cancer molecular subgroups. Ann Oncol. (2017) 28:1523–31. 10.1093/annonc/mdx156 28633480

[19] Wang Z Yang JJ Huang J Ye JY Zhang XC Tu HY. Lung adenocarcinoma harboring EGFR T790M and in Trans C797S responds to combination therapy of first- and third-generation EGFR TKIs and shifts allelic configuration at resistance. J Thorac Oncol. (2017) 12:1723–7. 10.1016/j.jtho.2017.06.017 28662863

[20] Dong J Zhu D Tang X Lu D Qiu X Li B. Circulating tumor cells in pulmonary vein and peripheral arterial provide a metric for PD-L1 diagnosis and prognosis of patients with non-small cell lung cancer. PLoS ONE. (2019) 14:e0220306. 10.1371/journal.pone.0220306 31348821 PMC6660086

[21] Okumura Y Tanaka F Yoneda K Hashimoto M Takuwa T Kondo N. Circulating tumor cells in pulmonary venous blood of primary lung cancer patients. Ann Thorac Surg. (2009) 87:1669–75. 10.1016/j.athoracsur.2009.03.073 19463575

[22] Frick MA Kao GD Aguarin L Chinniah C Swisher-McClure S Berman AT. Circulating tumor cell assessment in presumed early stage non-small cell lung cancer patients treated with stereotactic body radiation therapy: a prospective pilot study. Int J Radiat Oncol Biol Phys. (2018) 102:536–42. 10.1016/j.ijrobp.2018.06.041 30244877 PMC6154509

[23] Wendel M Bazhenova L Boshuizen R Kolatkar A Honnatti M Cho EH. Fluid biopsy for circulating tumor cell identification in patients with early-and late-stage non-small cell lung cancer: a glimpse into lung cancer biology. Phys Biol. (2012) 9:016005. 10.1088/1478-3967/9/1/016005 22307026 PMC3387995

[24] Liu YK Hu BS Li ZL He X Li Y Lu LG. An improved strategy to detect the epithelial-mesenchymal transition process in circulating tumor cells in hepatocellular carcinoma patients. Hepatol Int. (2016) 10:640–6. 10.1007/s12072-016-9732-7 27115761

[25] Si Y Lan G Deng Z Wang Y Lu Y Qin Y. Distribution and clinical significance of circulating tumor cells in nasopharyngeal carcinoma. Jpn J Clin Oncol. (2016) 46:622–30. 10.1093/jjco/hyw046 27162320

[26] Pardoll DM. The blockade of immune checkpoints in cancer immunotherapy. Nat Rev Cancer. (2012) 12:252–64. 10.1038/nrc3239 22437870 PMC4856023

[27] Borghaei H Brahmer J Horn L Ready N Steins M Felip E P2.35: nivolumab vs. docetaxel in advanced NSCLC: CheckMate 017/057 2-Y update and exploratory cytokine profile analysis: track: immunotherapy. J Thorac Oncol. (2016) 11:S237–S8. 10.1016/j.jtho.2016.08.106

[28] Horn L Spigel DR Vokes EE Holgado E Ready N Steins M. Nivolumab versus docetaxel in previously treated patients with advanced non-small-cell lung cancer: two-year outcomes from two randomized, open-label, phase III trials (CheckMate 017 and CheckMate 057). J Clin Oncol. (2017) 35:3924–33. 10.1200/JCO.2017.74.3062 29023213 PMC6075826

[29] Dong J Li B Lin D Zhou Q Huang D. Advances in targeted therapy and immunotherapy for non-small cell lung cancer based on accurate molecular typing. Front Pharmacol. (2019) 10:230. 10.3389/fphar.2019.00230 30930778 PMC6424010

[30] Dong J Li B Zhou Q Huang D. Advances in evidence-based medicine for immunotherapy of non-small cell lung cancer. J Evid Based Med. (2018) 11:278–87. 10.1111/jebm.12322 30444051

[31] Duruisseaux M Mc Leer-Florin A Moro-Sibilot D Cadranel J. Are ALK rearrangement variants promising predictive biomarker of ALK tyrosine kinase inhibitors efficacy? Ann Oncol. (2017) 28:1401. 10.1093/annonc/mdx116 28368436

