# Supplementary material for: Detection of Circulating Tumor Cell Molecular Subtype in Pulmonary Vein Predicting Prognosis of Stage I–III Non-small Cell Lung Cancer Patients
Source: Front Oncol. 2019 Oct 29;9:1139. doi: 10.3389/fonc.2019.01139 (PMC6830362; doi:10.3389/fonc.2019.01139)
Supplement: Supplementary file 1 [file Table_1.DOCX]

**supplementary Table S1. Baseline Clinical characteristics of mesenchymal CTC and non-mesenchymal CTC enrolled non-small cell Lung Cancer patients**

| **Characteristic** | **MCTC>ECTC (%)** | **MCTC≤ECTC (%)** | ***P* value** |
| --- | --- | --- | --- |
| Total patient numbers | 53/110 (48.2) | 57/110 (51.8) | 0.590 |
| Age(mean) | 60.0 | 59.4 | 0.883 |
| Gender |  |  | 0.364 |
| Female | 25/53 (47.2) | 22/57 (38.6) |  |
| Male | 28/53 (52.8) | 35/57 (61.4) |  |
| Smoking status(piece*year) | 349.05 | 392.9 | 0.136 |
| Histology |  |  |  |
| Squamous | 17/53 (32.1) | 23/57 (40.4) | 0.367 |
| Adenocarcinoma | 33/53 (62.2) | 32/57 (56.1) | 0.514 |
| Others* | 3/53 (5.7) | 2/57 (4.5) | 0.603 |
| Surgical method |  |  |  |
| Lobectomy | 39/53 (73.6) | 37/57 (64.9) | 0.325 |
| Segmentectomy | 8/53 (15.1) | 11/57 (19.3) | 0.560 |
| Sleeve lobectomy | 5/53 (9.4) | 7/57 (12.3) | 0.632 |
| Pneumonectomy | 1/53 (1.9) | 2/57 (4.5) | 0.602 |
| Stage(AJCC 8) |  |  | 0.096 |
| Stage I-II | 37/53 (69.8) | 31/57 (54.4) |  |
| Stage III | 16/53 (30.2) | 26/57 (45.6) |  |
| Performance status (EGOG) |  |  | 0.624 |
| 0-1 | 49/53 (92.5) | 54/57 (94.7) |  |
| 2 | 4/53 (7.5) | 3/57 (5.3) |  |
| Adjuvant Chemotherapy |  |  | 0.640 |
| Yes | 20/53 (37.7) | 24/57 (42.1) |  |
| No | 33/53 (62.3) | 33/57 (57.9) |  |
| Adjuvant Radiotherapy |  |  | 0.813 |
| Yes | 12/53 (22.6) | 14/57 (24.6) |  |
| No | 41/53 (77.4) | 43/57 (75.4) |  |
