# Supplementary material for: Detection of Circulating Tumor Cell Molecular Subtype in Pulmonary Vein Predicting Prognosis of Stage I–III Non-small Cell Lung Cancer Patients
Source: Front Oncol. 2019 Oct 29;9:1139. doi: 10.3389/fonc.2019.01139 (PMC6830362; doi:10.3389/fonc.2019.01139)
Supplement: Supplementary file 2 [file Table_2.DOCX]

**supplementary Table S2. Baseline Clinical characteristics of PD-L1(+) CTC and PD-L1(-) CTC enrolled non-small cell Lung Cancer patients**

| **Characteristic** | **PD-L1(+) CTC (%)** | **PD-L1(-) CTC (%)** | ***P* value** |
| --- | --- | --- | --- |
| Total patient numbers | 56/110 (50.1) | 54/110 (49.9) | 0.876 |
| Age(mean) | 60.2 | 61.7 | 0.659 |
| Gender |  |  | 0.008 |
| Female | 17/56 (30.4) | 30/54 (55.5) |  |
| Male | 39/56 (69.6) | 24/54 (44.4) |  |
| Smoking status(piece*year) | 394.8 | 350.6 | 0.344 |
| Histology |  |  |  |
| Squamous | 24/56 (42.9) | 16/54 (29.6) | 0.149 |
| Adenocarcinoma | 32/56 (57.1) | 34/54 (63.0) | 0.380 |
| Others* | 0/56 (0) | 4/54 (7.4) | - |
| Surgical method |  |  |  |
| Lobectomy | 39/56 (69.6) | 37/54 (68.5) | 0.898 |
| Segmentectomy | 8/56 (14.3) | 11/54 (20.4) | 0.399 |
| Sleeve lobectomy | 6/56 (10.7) | 6/54 (11.1) | 0.947 |
| Pneumonectomy | 3/56 (5.4) | 0/54 (0) | - |
| Stage(AJCC 8) |  |  | 0.881 |
| Stage I-II | 35/56 (62.5) | 33/54 (61.1) |  |
| Stage III | 21/56 (37.5) | 21/54 (38.9) |  |
| Performance status (EGOG) |  |  | 0.057 |
| 0-1 | 50/56 (89.2) | 53/54 (98.1) |  |
| 2 | 6/56 (10.8) | 1/54 (1.9) |  |
| Adjuvant Chemotherapy |  |  | 0.073 |
| Yes | 27/56 (48.2) | 17/54 (31.5) |  |
| No | 29/56 (51.8) | 37/54 (68.5) |  |
| Adjuvant Radiotherapy |  |  | 0.429 |
| Yes | 15/56 (26.8) | 11/54 (20.4) |  |
| No | 41/56 (73.2) | 43/54 (79.6) |  |
