# Supplementary material for: Detection of Circulating Tumor Cell Molecular Subtype in Pulmonary Vein Predicting Prognosis of Stage I–III Non-small Cell Lung Cancer Patients
Source: Front Oncol. 2019 Oct 29;9:1139. doi: 10.3389/fonc.2019.01139 (PMC6830362; doi:10.3389/fonc.2019.01139)
Supplement: Supplementary file 3 [file Table_3.DOCX]

**supplementary Table S3. Multivariate analyses of disease-free survival and overall survival (n=114)**

|  | Progression-free Survival | | |  | Overall Survival | | |
| --- | --- | --- | --- | --- | --- | --- | --- |
| Variables | HR | 95%CI | *P** |  | HR | 95%CI | *P** |
| Male vs. Female | 0.969 | 0.937-1/002 | 0.126 |  | 0.816 | 0.112-5.966 | 0.841 |
| Age | 1.396 | 0.423-4.608 | 0.584 |  | 1.021 | 0.963-1.082 | 0.486 |
| Index of smoke | 1.001 | 0.999-1.002 | 0.432 |  | 0.999 | 0.996-1.001 | 0.284 |
| Squamous vs. Other | 0.798 | 0.564-1.367 | 0.092 |  | 0.305 | 0.083-1.119 | 0.083 |
| Performance Status | 2.368 | 0.841-6.670 | 0.103 |  | 2.073 | 0.388-11.08 | 0.394 |
| EGFR+ vs. EGFR- | 1.103 | 0.451-2.702 | 0.830 |  | 0.613 | 0.162-2.326 | 0.472 |
| ALK+ vs. ALK- | 1.259 | 0.436-0.151 | 0.125 |  | 0.383 | 0.073-2.010 | 0.256 |
| BRAF+ vs. BRAF- | 2.337 | 0.572-9.547 | 0.237 |  | 2.311 | 0.332-16.08 | 0.397 |
| KRAS+ vs. KRAS- | 0.555 | 0.242-1.270 | 0.163 |  | 0.525 | 0.561-1.330 | 0.101 |
| ROS1+ vs. ROS1- | 1.287 | 0.399-4.149 | 0.672 |  | 0.441 | 0.085-2.286 | 0.329 |
| Stage I-II vs Stage III | 0.344 | 0.148-0.800 | 0.013 |  | 0.019 | 0.046-0.762 | 0.019 |
| Pulmonary vein CTCs | 0.274 | 0.112-0.671 | 0.005 |  | 0.398 | 0.100-1.580 | 0.190 |
| Adjuvant Chemotherapy | 0.814 | 0.292-2.273 | 0.695 |  | 0.975 | 0.238-3.999 | 0.972 |
| Adjuvant Radiotherapy | 1.722 | 0.632-4.692 | 0.228 |  | 1.000 | 0.203-4.932 | 1.000 |
| Mesenchymal vs. Other | 0.330 | 0.158-0.687 | 0.003 |  | 0.492 | 0.160-1.513 | 0.216 |
| PD-L1+ CTC vs -CTC | 0.475 | 0.205-1.097 | 0.081 |  | 1.687 | 0.424-6.716 | 0.458 |
| *Cox regression analysis was used to compare the statistical differences of various factors on survival | | | | | | | |
| Pulmonary vein CTC, CTC in pulmonary veins prior to lobectomy operation. | | | | | | | |
