# Supplementary figures and images for: Detection of Circulating Tumor Cell Molecular Subtype in Pulmonary Vein Predicting Prognosis of Stage I–III Non-small Cell Lung Cancer Patients
Source: Front Oncol. 2019 Oct 29;9:1139. doi: 10.3389/fonc.2019.01139 (PMC6830362; doi:10.3389/fonc.2019.01139)

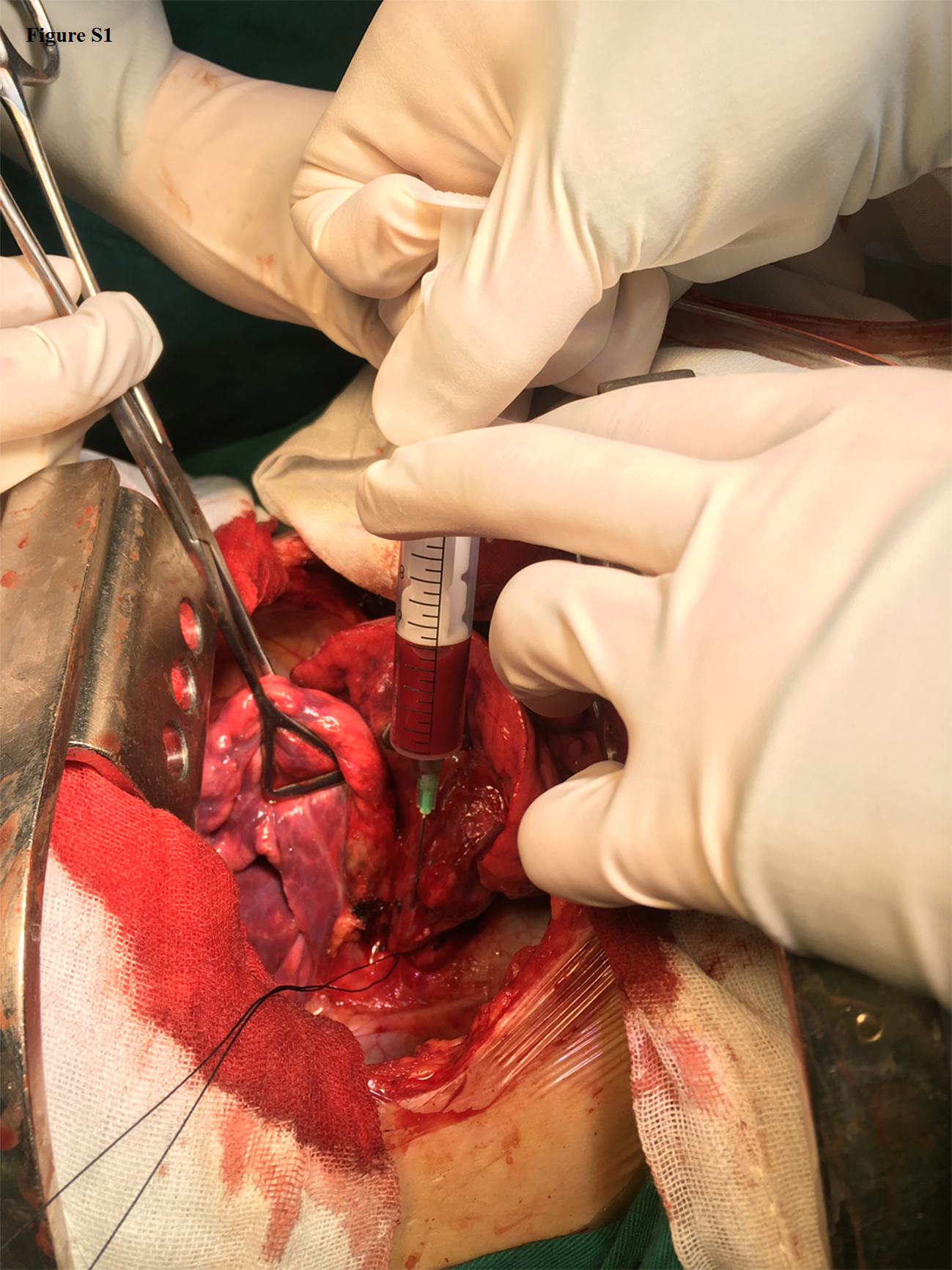

Supplement: Supplementary file 4 [file Image_1.tif]

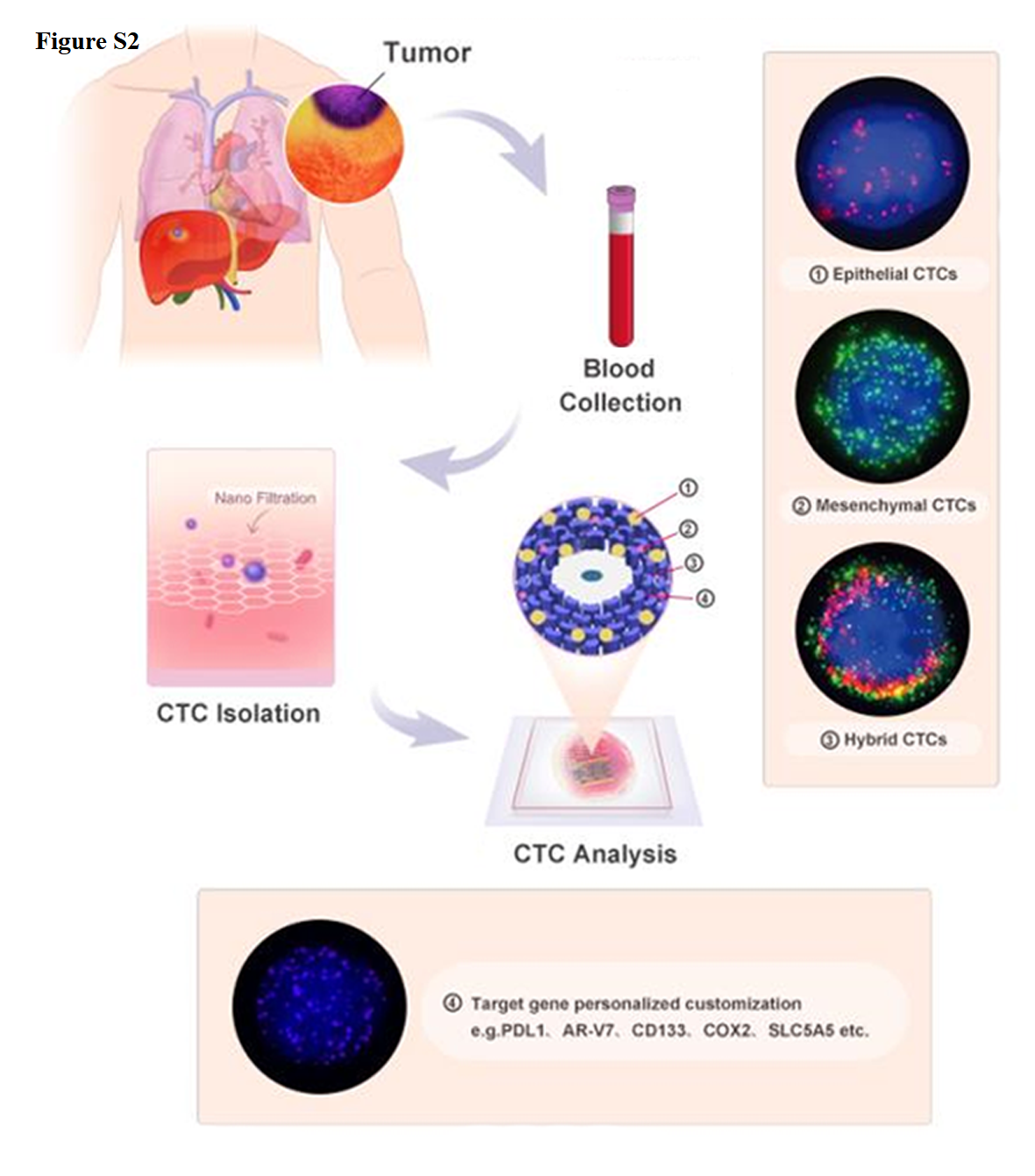

Supplement: Supplementary file 5 [file Image_2.tif]
